# The Th17 Lineage: From Barrier Surfaces Homeostasis to Autoimmunity, Cancer, and HIV-1 Pathogenesis

**DOI:** 10.3390/v9100303

**Published:** 2017-10-19

**Authors:** Vanessa Sue Wacleche, Alan Landay, Jean-Pierre Routy, Petronela Ancuta

**Affiliations:** 1 Département of Microbiologie, Infectiologie et Immunologie and Centre de Recherche du CHUM, Faculté de Médecine, Université de Montréal, Montréal, QC H2X 0A9, Canada; vanessa.sue.wacleche@umontreal.ca; 2 Department of Immunology and Microbiology, Rush University Medical Center, Chicago, IL 60612, USA; alan_landay@rush.edu; 3 Chronic Viral Illness Service and Division of Hematology, McGill University Health Centre, Montréal, QC H4A 3J1, Canada; jean-pierre.routy@mcgill.ca

**Keywords:** Th17 cells, CCR6, autoimmunity, cancer, HIV-1, gut, antiretroviral therapy

## Abstract

The T helper 17 (Th17) cells represent a subset of CD4+ T-cells with unique effector functions, developmental plasticity, and stem-cell features. Th17 cells bridge innate and adaptive immunity against fungal and bacterial infections at skin and mucosal barrier surfaces. Although Th17 cells have been extensively studied in the context of autoimmunity, their role in various other pathologies is underexplored and remains an area of open investigation. This review summarizes the history of Th17 cell discovery and the current knowledge relative to the beneficial role of Th17 cells in maintaining mucosal immunity homeostasis. We further discuss the concept of Th17 pathogenicity in the context of autoimmunity, cancer, and HIV infection, and we review the most recent discoveries on molecular mechanisms regulating HIV replication/persistence in pathogenic Th17 cells. Finally, we stress the need for novel fundamental research discovery-based Th17-specific therapeutic interventions to treat pathogenic conditions associated with Th17 abnormalities, including HIV infection.

## 1. History of Th17 Cell Discovery

The Th17 cells represent a major lineage of CD4+ T-cells producing the signature cytokine IL-17A that acts on epithelial cells, thus contributing to the maintenance of the first line of defense against pathogens at barrier surfaces [1,2,3,4]. The discovery of Th17 cells set the revision of the Th1/Th2 paradigm that was in place for more than 20 years [5,6]. The discovery of Th17 cells was precluded by the cloning in 2000 of a new cytokine chain called p19 able to associate with p40 chain of IL-12, thus solving controversies existent at that time in the field of autoimmunity [7]. Indeed, p19 and p40 form a new cytokine named IL-23 that is distinct from IL-12, which is formed by the p35 and p40 subunits [6]. The finding that antigen presenting cells (APC), such as dendritic cells (DCs), produce IL-23 and IL-12 in response to distinct pathogen-associated molecular patterns (PAMPs) further extended our understanding of the Th1 versus Th17 decision fate making. While microbial products such as lipopolysaccharides (LPS) and peptidoglycans (PGN) induce IL-12 production by DCs, other stimuli including anti-CD40 Abs, Adenosine Triphosphate (ATP), and PGE2 stimulation of DCs results in the production of IL-23 [8,9]. IL-23 binds to the IL-23R and IL-12Rβ1 chains, referred to as the IL-23R complex, whereas the IL-12 receptor consists of IL-12Rβ1 and IL-12Rβ2 chains [10]. It was initially believed that IL-12 and IL-23 complemented each other and acted at different steps during Th1 differentiation. In 2003, Aggarwal et al. reported that IL-23 acts on CD4+ T-cells and triggers the production of IL-17, a cytokine that was not expressed by Th1 and Th2 clones [11]. One month later, experiments performed by Cua et al. demonstrated that IL-23 but not IL-12 was responsible for the induction of experimental autoimmune encephalitis (EAE), a model of human multiple sclerosis (MS) [12]. A series of studies subsequently demonstrated that IL-17-producing cells, driven by IL-23, were responsible for the induction of EAE [13,14]. Interestingly, cells producing IL-17 were demonstrated to confer protection against extracellular pathogens such as *Klebsiella pneumonia* [15]. In 2005, the scientific community acknowledged the existence of a new subset of CD4+ T-cells distinct from Th1 and Th2, a subset that is now referred to as Th17 cells [16,17]. At this time, many other T-cell lineages have been described including regulatory T cells (Tregs), follicular helper T-cells (Tfh), as well as IL-9 (Th9), and IL-22-producing T-cells (Th22) [18,19,20,21,22]. It is to be anticipated that the constantly evolving technological advances will allow future identification of other novel T-cell lineages with specific functions in immunity and disease pathogenesis.

## 2. Role of Th17 Cells in Promoting Immunity at Barrier Surfaces

Th17 cells play an important role in the induction of protective immunity against bacterial and fungal infection at mucosal sites such as the gut, lung, and the oral cavity [23,24,25]. The number of Th17 cells in humans and mice is small (relative to Th1 cells) under non-pathological conditions [26,27]. Murine Th17 cells under steady state reside in the intestine where they are generated due to the presence of specific members of the commensal microbiota, including the segmented filamentous bacteria (SFB) [28,29,30]. SFB promote the production of serum amyloid A (SAA) and ATP, activating the lamina propria APCs to induce Th17 differentiation [28,31,32]. The Th17-associated protective functions involve the secretion of several cytokines including IL-17A, IL-17F, IL-21, IL-22, IL-26, IL-8, and CCL20 [4].

### 2.1. IL-17A

IL-17A signals through its receptors IL-17RA and IL-17RC that are mostly expressed in non-hematopoietic cells such as the epithelial and mesenchymal cells [4]. The IL-17 receptors include conserved cytoplasmic motifs termed SEF/IL-17R (SEFIR) that interact with adaptor protein ACT1, activating downstream the NF-κB and MAPK pathways. One important role of IL-17A signaling is the regulation of intestinal epithelial cell permeability [33,34]. In addition, IL-17A signaling in epithelial cells induces the production of the chemokines CXCL1, CXCL2, CXCL5, and CXCL8/IL-8, which will result in recruitment of neutrophils. In addition, IL-17A leads to the production of CCL20 that promotes the recruitment of CCR6−expressing cells such as Th17 cells [35]. Th17 cells themselves produce CCL20 and CXCL8, attracting even more Th17 lymphocytes and neutrophils, respectively, at inflammatory sites [4,36]. Downstream IL-17A effector molecules also include IL-6, TNF-α, and GM-CSF that regulate the biological functions of myeloid cell lineages, especially neutrophils. Interestingly, IL-6 acts in a positive feedback loop, amplifying Th17 differentiation. Furthermore, IL-17A is an inducer of antimicrobial peptides including the β-defensins and lipocalin 2 (LCN2) that prevent infection at mucosal surfaces [4,37]. Although IL-17A is a weak activator of signaling pathways, its activity is increased when combined with other cytokines/factors including TNF-α, IL-6, IL-22, IL-1β, IFN-γ, CD40, and LPS, for the regulation of target genes. The complete molecular basis explaining this synergy is still not well understood. However, studies suggest that the mechanism of synergy involves increase in the expression of IL-17R and stabilization of the induced cytokines mRNA [35,38]. Another function of IL-17A is the activation of B-cell germinal center formation and antibody responses [39].

### 2.2. IL-17F

IL-17F shares ~50% homology with IL-17A, and both have overlapping functions [40]. However, IL-17F is less potent than IL-17A in the induction of downstream signaling via IL-17RA/RC. Interestingly, cell fate mapping experiments demonstrated that the IL-17F but not the IL-17A homodimer is highly expressed in naive T-cells differentiating towards the Th17 cell-line [41,42]. IL-17F is thus considered as an early marker of Th17 differentiation. Interestingly, patients with chronic mucocutaneous candidiasis were shown to exhibit defects in IL-17F and IL-17RA but not IL-17A, indicating that IL-17F has a defined protective role against this infection [43].

### 2.3. IL-22

IL-22, similar to IL-17A, induces production of antimicrobial peptide from epithelial cells and contributes to epithelial cell proliferation, survival, and tissue repair in the intestine [23,44]. IL-22 provides protection against hepatitis and inflammatory bowel disease (IBD) [45,46,47]. IL-22 has however a dual role in immunity as it can induce inflammation as well [48]. In this sense, IL-22-stimulated keratinocytes produce pro-inflammatory molecules that promote psoriasis [49].

### 2.4. IL-26

IL-26 is a cytokine specifically produced by Th17 cells with a role that remains underexplored. Very recent studies demonstrated that IL-26 is an antimicrobial factor that mediates sensing of bacterial and host-cell DNA [50]. Through IL-26, Th17 cells can kill extracellular bacteria by inducing pores in cell membrane. Furthermore, IL-26 can interact with the bacterial and self-genome which will lead to production of type 1 IFN by plasmacytoid DCs (pDCs). IL-26 was identified and mainly studied in humans [51]. Therefore, the role of IL-26 remains to be examined in more detail in murine models.

The mechanism of action of Th17 cells is pathogen-specific. For example, Th17 immune response against *Klebsiella pneumonia*, mediated by IL-17A and IL-22 includes the induction of CXCL1, CXCL2, CXCL5 causing neutrophil infiltration and the production of lipocalin-2 (LCN2), an enzyme subsequently restricting bacterial growth by preventing access to dietary iron [37]. On the other hand, induction of β-defensins 1, 2, 3, and 4 by Th17 cells is sufficient for the clearance of *Citrobacter rodendium*, infecting the gut mucosa. Interestingly, vaccine-induced responses to *Mycobacterium tuberculosis* reveal that IL-17A recruits Th1 cells via the regulation of CXCR3 ligands CXCL9, CXCL10, and CXCL11 [52]. The list of pathogens inducing Th17-specific responses also includes *C. albicans*, *S. aureus*, *Salmonella enterica*, *Escherichia coli*, *Helicobacter pilori*, *Bordetella pertussis*, *Blastmomyces dermatitidis*, *Coccidioides posodasii*, *Porphyromosnas gingivalis*, *Listeria monocytogenes*, *Pneumocystis carinii*, *Aspergillis fumigatus,* and *Toxoplasma gondii* [44,53,54].

In conclusion, Th17 cells act at the interface between innate and adaptive immunity at barrier surfaces. Although the role of Th17 cells in mediating the cognate immunity against various pathogens is increasingly documented [55], their role in innate immunity by maintaining the integrity of mucosal epithelial barriers is extremely well documented. Indeed, Th17-specific cytokines act directly on epithelial cells to maintain a physical and biological barrier that prevents the process of microbial translocation from the gut, a deleterious process that occurs in the context of Th17 cell quantitative and/or qualitative dysfunction [1,56,57,58,59,60]. In parallel, epithelial cells interacting with the local microbiota regulate the differentiation and function of Th17 cells [28,29,32,61,62,63].

## 3. Transcriptional Regulation of Th17 Differentiation

### 3.1. Positive Regulators

A subset of T-cells can be considered a distinct lineage when it expresses specific effector functions under the control of unique transcriptional regulators [55,64].

#### 3.1.1. Retinoic Acid-Related Orphan Receptor (ROR) Gamma t (RORγt)

Shortly after the acknowledgement of Th17 existence [16,17], the master regulator of Th17 lineage polarization, RORγt, was identified [65,66]. Two different distinct approaches led to this identification. The first approach consisted in the use of engineered mice with a GFP reporter knocked-in into the RORγt gene; only cells positive for GFP were shown to express IL-17A [66]. Mice deficient for *rorc*, the gene encoding RORγt, could not generate Th17 cells and showed diminished susceptibility to EAE [66]. Conversely, RORγt ectopic expression in naive T-cells leads to expression of Th17 lineage-specific cytokines. The second approach involved genome-wide transcriptional profiling of activated cells differentiated into Th17 versus Th1 cells [65]. The RORγt is expressed in mice, whereas its homologue RORC2 is the master regulator for human Th17 cells [67]. The identification of RORC mutations in humans provided the final proof on the role played by this transcription factor in Th17 differentiation and mucosal immunity against pathogens such as *Candida* and *Mycobacterium* [68]. In addition to RORγt/RORC2, other transcription factors were subsequently reported to play essential roles in the development of Th17 cells, including STAT3, Basic leucine zipper transcription factor ATF-like (BATF), interferon regulatory factor 4 (IFR4), aryl hydrocarbon receptor (AhR), and RORα [69,70,71,72,73,74]. Most recently, the double susceptibility to *Candida albicans* and *Mycobacterium* species was associated with RORC mutations in humans [68]. Finally, RORα was also shown to interact with RORγt [74]. From the four identified isoforms of RORα, only RORα4 has been characterized in the regulation of Th17 differentiation [74]. The lack of functional RORα in T-cells leads to an impairment in IL-17A production. RORα was reported to synergistically act with RORγt for increased IL-17, IL-17F, and IL-23R expression. The distinct role of RORα in mice and humans regarding Th17 development is not clearly understood. Determining whether RORα and RORγt have functional redundancy in terms of binding to distinct or identical promoter elements and promoting overlapping or different gene expression in T-cells requires further investigation.

#### 3.1.2. Signal Transduction and Activation of Transcription 3 (STAT3)

STAT3 is one of the first transcription factors up-regulated within the first hours of Th17 polarization [75]. Using chromatin immunoprecipitation followed by sequencing (CHIP-seq), Durant et al. demonstrated that STAT3 directly regulates the transcription of *il17a*, *il17f*, and *il23r* genes and controls the expression of RORC, BATF, and IRF4 via epigenetic modifications [76]. This is consistent with previous findings that STAT3 increases RORγt expression [66]. The link between STAT3 expression and IL-17A and IL-17F production was also demonstrated in humans in the context of autosomal dominant hyper-IgE syndrome (HIES, also called Job’s syndrome) [77,78]. The HIES is characterized by a dominant negative mutations in *stat3* gene, resulting in the deficiency of Th17 lymphocytes and impaired host defense against pathogens controlled by Th17 immune responses including *Candida albicans* and *Staphylococcus aureus* [79].

#### 3.1.3. Basic Leucine Zipper ATF-Like Transcriptional Factor (BATF)

BATF belongs to the FOS-like AP1 family of transcription factors and acts as a positive regulator for RORγt, IL-17, and IL-23R expression [4]. Although mice deficient for BATF express similar levels of RORγt compared to wild-type (WT) at early stages of Th17 differentiation, RORγt expression is only temporary and is not maintained for complete Th17 development in the absence of BATF [70]. Partial restoration of IL-17-producing cells in BATF-deficient animals was observed upon overexpression of RORγt, suggesting that BATF acts upstream RORγt to drive Th17 differentiation [70]. BATF dimerizes with the transcription factor AP-1 and binds to the promoter regions of the *il17a*, *il21*, and *il22* genes [70].

#### 3.1.4. Interferon Regulatory Factor 4 (IRF4)

Originally described as a Th2 regulator, IRF4 was found to contribute to Th17 development [71]. Mice deficient in IRF4 showed impaired Th17 differentiation and resistance to EAE and this despite normal RORγt expression. Similar to BATF, overexpression of RORγt in IRF4-deficient mice partially restored Th17 development, indicating that RORγt and IRF4 cooperatively control Th17 differentiation [71]. Ciofani et al. observed that BATF and IRF4 function as pioneer transcription factors, governing initial chromatin accessibility that allows RORγt recruitment and interaction with Th17-relevant genes [80]. In fact, STAT3 forms a complex with BATF and IRF4, resulting in the recruitment of RORγt on the promoter of specific genes thus up-regulating Th17-related genes while suppressing the fate of Th1 and Th2 lineages [80]. The transcription factors included in the complex described above regulate DNA-binding activities of each other and together control cell fate commitment [4,81]. Therefore, RORγt is major but not the sole regulator of Th17 development. The association between BATF and IRF4 occurs only in TCR-activated cells [80].

#### 3.1.5. Aryl Hydrocarbon Receptor (AhR)

Dependent on its interaction with a specific agonist, AhR acts as a booster for Th17 differentiation [72,73]. Also, the presence of natural AhR agonists in the culture media was demonstrated to be important for optimal Th17 differentiation in vitro [82]. Although AhR-deficient mice still develop Th17 cells, they lack the ability to produce IL-22 [73]. The findings that AhR represents a molecular signature for non-pathogenic Th17 cells in mice [83] and humans [84] and is critical for the transdifferentiation of Th17 cells into Tregs during the resolution of inflammation [85], adds a new level of complexity to the role of AhR in the control of Th17 differentiation and function.

#### 3.1.6. Other Transcription Factors Involved in Th17 Polarization

Further, other transcription factors were found to be positive regulators of Th17 differentiation; this includes c-Maf, SRY-related high-mobility-group-box (Sox5), Aiolos, Ikaros, IκBζ, inhibitor of nuclear factor-κB kinase-α (IKKα), RUNX1, hypoxia-inducible factor alpha (HIFα), and promyelocytic leukemia zinc finger protein (PLZ) [86,87,88]. Apparently, C-maf is required for the maintenance of the Th17 lineage and the production of IL-21 [89,90]. Tanaka et al. demonstrated that in combination with Sox5, c-Maf induces Th17 differentiation downstream of STAT3 [86]. Aiolos is up-regulated by STAT3 and Ahr following Th17 polarization [91]. Aiolos promotes Th17 differentiation by silencing the *il2* locus, and IL-2 was found to inhibit Th17 development [92,93]. Ikaros prevents repressive chromatin modifications of *ahr*, *rorc*, *il17a*, and *il22*, thereby promoting Th17-related gene expression upon polarizing cues [94]. Also, Ikaros represses T-bet and the regulatory T-cell (Tregs)-specific transcription factor Foxp3. The IκBζ, is encoded by the *Nfkbiz* gene and is up-regulated following IL-6 and TGF-β stimulation [95]. The IκBζ interacts with RORγt and RORα, increasing IL-17 production. The Nfkbiz-deficient mice are resistant to EAE due to a defect in Th17 differentiation. IKKα binds to the *il17a* locus and subsequently promotes gene transcription leading to IL-17A production upon Th17 polarization [96]. The non-activated form of IKKα leads to reduced frequency of IL-17A producing lymphocytes. RUNX positively regulates Th17 lineage by inducing RORγt expression, interacting with this master regulator and the promoter region of *il17a* [97]. HIFα functions as a sensor to hypoxia; it is up-regulated under hypoxic conditions and IL-6 signaling pathways involving STAT3 [4,98]. HIFα interacts and promotes *rorc* gene transcription and associates with RORγt to drive *il17a* transcription [98]. PLZ was demonstrated to be important for Th17 differentiation and in the maintenance of CCR6 expression at the surface of Th17 cells [88]. Finally, the protein deacetylase Sirtuin 1 (SIRT1) was demonstrated to deacetylate RORγt and this way to boost Th17 effector functions [99].

### 3.2. Negative Regulators

Lineage-specific transcription factors such as T-bet (for Th1) and FOXP3 (for Tregs) have been reported to suppress Th17 development. T-bet suppresses the Th17 lineage by inhibiting IRF4 expression and by competing with RORγt for RUNX1 binding [100,101]. FOXP3 directly binds to RORγt to prevent Th17 differentiation [102]. It was also found that FOXP3 binding to RUNX1 prevents Th17 development [97]. Other Th17 negative regulators include: T-cell factor 1 (TCF-1), Growth factor independent 1 (GFI-1), Interferon regulatory factor 8 (IRF8), Twist Family BHLH Transcription Factor 1 (TWIST1), peroxisome proliferator-activated receptor gamma (PPARγ), E-twenty six 1 (ETS1), E74-like factor 4 (ELF4), inhibitor of DNA-binding 3 (ID3), and Early growth response gene (EGR2) [87]. TCF-1 interacts with the *il17a* locus, leading to gene silencing [103]. TCF-1 also inhibits IL-7R expression, affecting Th17 survival capacity. GFI-1 suppresses Th17-related gene expression to promote Th2 differentiation [104], while IRF8 interacts with RORγt to shut down Th17 development [105], but the complete mechanism used by IRF8 to suppress Th17 differentiation is still unknown. TWIST1 is induced following activation of the IL-6-STAT3 signaling pathways and acts as a regulator of Th17 differentiation by repressing *il6ra* gene expression [106]. TWIST1-deficient mice exhibit higher frequency of Th17 cells. Activation of PPARγ prevents the removal of repressor on the promoter region of RORγt [107], inhibiting Th17 differentiation without affecting Th1 or Th2 lineage development. In mice, ETS1 was shown to favor IL-2-STAT5 axis, shutting down Th17 differentiation [108]. Another member of the ETS transcription factor family, ELF4, was also shown to regulate Th17 development in a different manner than ETS [109]. The IL-6 and TGF-β signaling threshold to initiate Th17 differentiation was decreased in mice deficient in ELF4. Through unknown mechanisms, ID3 also represses Th17 differentiation as demonstrated by studies in ID3-deficient animals that expressed increased frequencies of Th17 cells [110]. EGR2 interaction with BATF prevents the formation of the transcriptional complex involved in the initiation of Th17 differentiation [111].

Most of the studies cited above were performed using mouse models. In human cells, transcriptional regulators shown to be involved in Th17 development include the positive regulators AHR, IRF4, c-maf, BATF, and PLZ [80,82,84,87,88,112] and the negative regulators TWIST1, PPARγ, and EGR-2 [106,107,111].

In conclusion, the modulation of Th17 development is tightly regulated and involves a set of transcription factors with specific functions at different levels [4,87]. Molecular mechanisms involved in the regulation of Th17 polarization are illustrated in multiple reviews published previously by other groups [4,113,114].

## 4. Cytokines Involved in Th17 Lineage Polarization

IL-23 was originally described as the key inducer of pathogenic CD4+ T-cells producing IL-17A in EAE murine models [13,14]. However, it was later found that IL-23 alone does not drive Th17 differentiation but rather has a role in the lineage expansion, maintenance, and survival [115,116,117]. This observation indicated that other components are involved in the induction of IL-17-producing cells from naive precursors. Several laboratories including the groups of Stockinger, Weaver, Kuchroo, and Littman demonstrated that the presence of both TGF-β and IL-6 in the context of antigenic presentation promoted murine Th17 development [116,117,118,119]. IL-6 signaling activates STAT3, which subsequently drives Th17 differentiation [69]. Mice deficient in IL-6 are unable to generate Th17 cells and are resistant to EAE [4]. Consistently, IL-6 is currently being used as a target for treatment against RA and other inflammatory conditions. The need of TGF-β for Th17 differentiation remains controversial. Experiments involving inhibition of the TGF-β signaling complex and deletion of TGF-β gene on T-cells indicate that TGF-β is essential for Th17 development in mice [120,121,122]. Nevertheless, deletion of TGF-β led to the substantial increase of IFN-γ and IL-4 production, suggesting that TGF-β shuts down Th1 and Th2 differentiation pathways [120,121], allowing for Th17 development. In the absence of TGF-β, IL-6 alone can promote Th17 differentiation in cells deficient for T-bet and STAT6 [123], indicating that TGF-β indirectly induces Th17 development by restraining T-bet and GATA3 expression. The controversial requirement of TGF-β in Th17 differentiation was further highlighted in human studies. Indeed, several groups demonstrated that a combination of IL-6, IL-1β, and IL-23 is sufficient for the development of human Th17 cells [124,125,126] and that the requirement of TGF-β is not essential [65,127]. Similar findings were also reported in mice [128]. In contrast, other researchers reported that TGF-β was essential for human Th17 differentiation [129,130,131], especially for the induction of T-cells producing homogenously IL-17A [130]. In fact, the absence of TGF-β led to Th1Th17 generation [130]. Other studies reported that the addition of TGF-β was needed for up regulation of RORγt expression but not IL-17A production [129,132]. Some researchers argue that it is difficult to exclude the importance of TGF-β in experiments involving in vitro induction of Th17 cells from naive precursors since low levels of TGF-β are known to be present in serum [129]. TGF-β was previously shown to be a key factor in the generation of inducible Treg (iTreg) [133]. The involvement of TGF-β in the development iTregs and Th17 cells appears to be counterintuitive. The current understanding is that high doses of TGF-β promote expression of FOXP3 that will repress RORγt, whereas low doses of TGF-β combined with IL-6 overcome FOXP3 repression of RORγt [3]. The combination of TGF-β and IL-6 does not induce pathogenic Th17 cells [4]. Subsequent cell exposure to IL-23 drives pathogenicity in Th17 cells [128,134,135]. The IL-1β signaling is critical in Th17 differentiation as revealed by studies in IL-1 receptor-deficient mice that fail to generate antigen-specific Th17 cells and are protected from EAE [136]. The expression of IL-R1 is induced in Th17 cells by IL-6, and signaling through this receptor leads to the expression of IRF4, which will strengthen RORγt function [137]. IL-1β is important for the phosphorylation of mammalian targets of rapamycin (mTOR) that will increase the metabolic fitness of newly dividing Th17 cells under inflammatory conditions [138]. Therefore, the presence of IL-1β is important for the expansion of the Th17 lineage competing with other lineages in a hostile cytokine environment. Pertussis toxin-mediated IL-1β production by myeloid cells is essential for the development of EAE [139]. IL-21 is another important cytokine for the expansion of Th17 cells [90]. In fact, IL-21 represents a key survival factor for Th17 cells [140] and may compensate for the intrinsic inability of Th17 cells to produce IL-2 [141]. Studies by Pallikkuth et al. demonstrated that supplementation with IL-21 contributes to the restoration of Th17 cells in a simian model of AIDS [142]. IL-21 is recognized as a key marker for follicular helper T-cells (Tfh) [22,143], and thus IL-21 being produced in autocrine manner by Th17 cells with Tfh features remains one possibility [144].

## 5. Th17 Lineage Plasticity

The Th17 lineage plasticity is described as the ability of Th17 cells to acquire new effector features, while losing their original identity as defined by the expression of RORγt and IL-17A [4]. The plasticity of Th lineages depends on epigenetic modifications dictating the expression or repression of lineage-specific transcription factors [145,146,147]. The epigenetic modification basically consists of permissive (H3K4me3) versus repressive (H3K27me3) histone marks [4]. Although Th1 and Th2 cells are enriched in repressive histone marks for the *rorc* and *il17a* loci, Th17 cells exhibit both permissive and repressive marks on *tbet* and *gata3* [148]. This observation suggests that Th17 cells have the capacity to acquire Th1 or Th2 features when exposed to specific polarization stimuli within their environment. Indeed, the plasticity of Th17 cells towards the Th1 program is well documented [3,4,149]. Studies performed in mice and humans demonstrated the down-regulation of RORγt, IL-17A, IL-17F, IL-22, and CCR6 as well as the up-regulation of T-bet and IFN-γ in Th17 cells when cultured in the presence of IL-12 [150,151,152,153,154]. Th17 cells exposed to IL-12 co-express T-bet and RORγt, similar to IL-17A+IFN-γ+ double positive cells (Th1Th17 profile). These Th17 cells with Th1 features are referred to as Th1Th17 [155] or most recently Th1* cells [68,156], as mentioned above. Fate-mapping reporter mice experiments demonstrated that IL-17A-producing cells acquire the ability to co-express IFN-γ under EAE-associated inflammatory conditions; this shift was mainly mediated by IL-23 [157]. In humans, epigenetic studies also suggested that Th1Th17 cells originate from Th17 cells [158]. Nevertheless, other findings using TCRβ deep sequencing techniques in the context of antigenic presentation indicate that a fraction of Th1Th17 cells may originate directly from naive precursors without any transition through an initial Th17 stage [156]. Similar to IL-12 and IL-23, IL-1β was found to induce IFN-γ in Th17 cells. Zielinski et al. demonstrated that *C. albicans* but not *S. aureus* generated IL17A+IFNγ+ populations in humans Th17 cells and that priming of IL-17A and IFN-γ double-producing cells was mediated by IL-1β [159]. Recently, TNF-α was also found to drive the shift of Th17 cells towards the Th1/Th17 subsets [160].

The Th17 plasticity is not limited only toward the Th1 axis, as Th17 cells were also shown to shift toward other lineages, including Th2, Tfh, and Tregs [161,162,163]. For example, memory CCR6+CD161+ Th17 cells exposed to a rich IL-4 microenvironment acquired the ability to produce Th2 lineage-specific cytokines including IL-4 and IL-5 while maintaining their ability to express IL-17A, IL-21, and IL-22 [161]. These cells were referred to as Th17/Th2 cells and were detected in the peripheral blood of patients with chronic asthma. The Tfh development is still controversial and one of the proposed models involve the concept that a naive CD4+ T-cell initially undergoes a Th1, Th2, or Th17 differentiation program before becoming a Tfh lymphocyte [22]. In line with this model, fate-mapping reporter mice experiments demonstrated the shift of Th17 cells acquiring Tfh properties and thereby becoming able to help B-cell produce IgA [162]. Although the shift of Treg towards the Th17 progeny has been observed [164,165], only few studies found the ability of Th17 cells to transition into iTreg [85,163]. This conversion was dependent on a specific type of monocyte being able to produce high levels of TGF-β and retinoic acid [163]. Most recent studies support indeed the ability of Th17 cells to trans-differentiate into Tregs, a process that occurs naturally during the resolution of inflammation [85].

## 6. Surface Markers Defining Human Th17 Subsets

Pioneering studies by Sallusto, Lanzaveccia et al. demonstrated that chemokine receptors are markers for T-cell subsets with distinct functional features and antigenic specificities [55]. In 1997, the chemokine receptor CCR3 was identified as preferentially expressed on Th2 cells [166]. Two years later, this same group reported that differential expression of CCR7, a chemokine receptor mediating trafficking into lymph nodes [167], together with CD45RA, a receptor-linked protein tyrosine phosphatase [168], identify subsets of CD4+ and CD8+ T-cell subsets with naive (CCR7+CD45RA+), central memory (CCR7+CD45RA-), and effector memory features (CCR7-CD45RA-) [169]. In 2004, CXCR3 and CCR4 chemokine receptors were further reported to identify CD4+ T-cell lineages with distinct polarization profiles and antigenic specificities: CXCR3 for Th1 cells and CCR4 for Th2 cells [170]. The CXCR3 mediates cell migration into inflammatory sites, whereas CCR4 is a skin-homing marker [171,172]. In 2007, two years after the official identification of human Th17 cells [16,17], the chemokine receptor CCR6 was identified as the main surface marker characterizing the Th17 lineage [152,155,173]. Murine Th17 cells were found to express CCR6 as well [174]. CCR6 regulates Th17 trafficking into the gut [175] and other anatomic sites including the brain [176]. Differential expression of CCR4, CCR6, and CXCR3 distinguishes functionally distinct Th17 subsets. The co-expression of CCR6 and CCR4 identifies human Th17 cells that produce IL-17A and proliferate in response to *C. albicans* and *S. aureus* [155,159]. The co-expression of CCR6 and CXCR3 identifies a heterogeneous population, known as Th1Th17 that produces both IL-17A and IFN-γ [155]. Precisely, Th1Th17 cells, also known as non-classical Th1 cells or Th1*, express RORC as well as T-bet and comprise a major fraction of Th1 cells producing IFN-γ and a minor population of cells co-expressing IL-17A and IFN-γ [24,155,156]. Th1Th17/Th1* cells were originally found to mediate immune responses against *M. tuberculosis* but were later reported to proliferate in response to *C. albicans* and *S. aureus* as well [155,156,177]. Both human Th17 and Th1Th17/Th1* cells express IL-23R, IL-1R, CD26 and CD161 at their surface [125,178]. Originally found to be expressed in natural-killer (NK) and NKT cells, the C-type lectin CD161 was also shown to identify Th17 precursors [179]. As opposed to Th17 lymphocytes, Th1Th17/Th1* cells express at their surface the IL-12 receptor [24], suggesting their ability to respond to this Th1-polarizing cytokine. The fact that Th1Th17/Th1* share features of Th17 and Th1 cells is consistent with the existence of a developmental relationship between the Th17 and Th1 lymphocytes [55,149,180].

**Figure 1:**
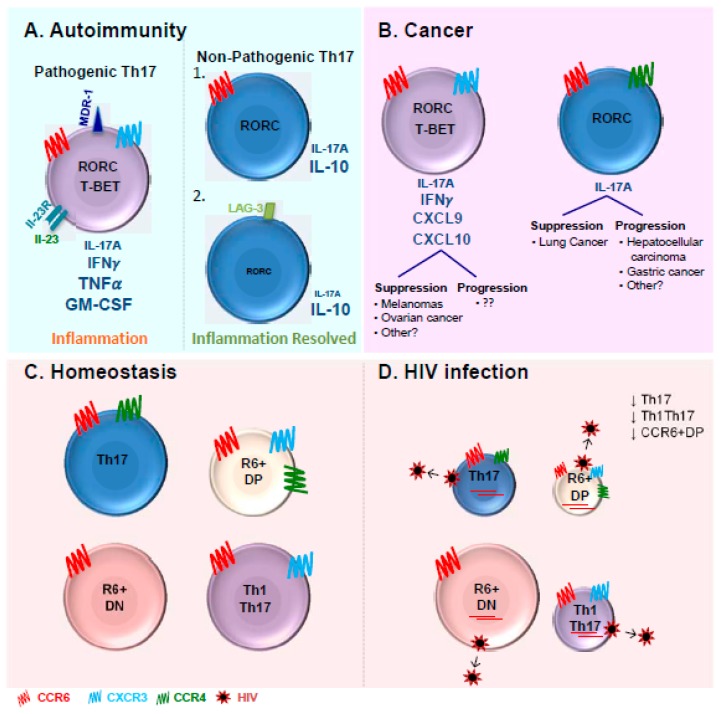
The Th17 lineage heterogeneity during homeostasis and disease pathogenesis. This figure summarizes the heterogeneity of Th17 cells as discovered in mice and/or humans. (**A**) The concept of pathogenic versus non-pathogenic Th17 cells was originally established in the context of autoimmunity. Pathogenic Th17 cells are characterized by their ability to produce pro-inflammatory cytokines such as IL-17A, TNF-α, GM-CSF, and IFN-γ. These cells are identified as Th1Th17 or Th1* cells. They express on their surface the drug efflux pump MDR-1 and the receptor for IL-23, a cytokine found to promote pathogenic Th17 cell features. At the opposite, non-pathogenic Th17 cells are characterized by their ability to produce the immune-suppressive cytokine IL-10 or to express LAG-3; (**B**) the long-lived properties of certain Th17 subsets raised their potential contribution to cancer progression. However, the role of Th17 cells remains controversial and appears to vary from beneficial to deleterious depending on the type of cancer. Future studies are required to clarify these differences; (**C**,**D**) in humans, four subsets of CCR6+ Th17 cells were identified by our group based on their differential expression of CCR4 and CXCR3, as follows: CCR4+CXCR3− (Th17), CCR4−CXCR3+ (Th1 Th17 or Th1*), CCR4−CXCR3− (double negative, CCR6+DN), and CCR4+CXCR3+ (double positive, CCR6+DP). In HIV-uninfected individuals (**C**), Th17, Th1Th17, and CCR6+DN subsets are present in the blood at similar frequency, whereas CCR6+DP cells are less predominant. In chronically HIV-infected individuals receiving viral suppressive antiretroviral therapy (ART) (**D**), the frequency of Th17, Th1Th17, and CCR6+DP is dramatically decreased. However, the frequency of CCR6+DN cells is preserved in the blood and this subset is the most predominant in the lymph nodes of ART-treated individuals.

Our group extended the understanding of Th17 biology by recently identifying two previously uncharacterized Th17 subsets co-expressing or lacking the chemokine receptors CXCR3 and CCR4, termed CCR6+ double negative (CCR6+DN; CXCR3-CCR4-) and CCR6+ double positive (CCR6+DP; CXCR3+CCR4+) cells [181] (Figure 1). Following TCR triggering, the CCR6+DN cells produced IL-17A at levels similar to those observed in the Th17 cells, whereas the CCR6+DP cells expressed low levels of IL-17A comparable to those produced by Th1Th17/Th1* cells. Both populations expressed considerate levels of IFN-γ. Similar to Th17 and Th1Th17/Th1* cells, the CCR6+DN cells proliferated in response to *C. albicans*. Transcriptional profiling revealed that the CCR6+DN cells express markers of early Th17 development (IL-17F, STAT3), lymph-node homing (CCR7, CD62L), follicular help (CXCR5, BCL6, ASCL2), and self-renewal (LEFI, MYC, TERC), and preferentially produce IL-17F, IL-21, and IL-8 upon TCR triggering in vitro [181]. This discovery raises new questions on the differentiation relationship between these four Th17 subsets and their differential implication in disease pathogenesis.

The use of surface markers for the identification of Th17 subsets is instrumental for immune monitoring to study disease pathogenesis and measure responses to treatment. However, the fact that not all CCR6+ T-cells produce Th17 effector cytokines raises questions regarding the possibility of overestimating the frequency of Th17 cells by using surface markers. Nevertheless, the group of Dan Littman recently provided experimental evidence that improved our current understanding of Th17 polarization as a process including at least two major steps: (i) specification of polarizing profile, and (ii) induction of effector functions, [32]. This supports the idea that Th17 identification based on effector functions (i.e., IL-17A production) leads to the underestimation of Th17 frequency. Indeed, early studies by Unutmaz et al. demonstrated that sorted CCR6+IL-17A− T-cells acquire the ability to produce IL-17A upon signaling via specific cytokines in vitro (i.e., IL-2, IL-7, IL-15) [182]. Similarly, the expression of the Th17 master regulator RORγt is not constitutive but requires TCR triggering (personal observations), thus limiting the possibility of using functional markers for the identification of Th17 cells ex vivo. Finally, a recent study investigated the dynamics of Th17 cells in HIV-infected individuals by using in parallel phenotypic and functional markers; the authors reached the conclusion that both approaches are valid as they lead to identical scientific conclusions [183].

The majority of human studies listed above were performed with peripheral blood Th17 cells. Nevertheless, studies performed on cells infiltrating peripheral tissues in humans including the intestinal or vaginal mucosa also revealed a functional heterogeneity among CD4+ T-cell subsets in terms of Th17 and Th1Th17 polarization [184,185,186,187,188,189,190,191]. Emerging evidence exists now supporting the ability of specific components of intestinal microbiota in shaping the functional heterogeneity of CD4+ T-cells, including Th17 fate decision [28,29,32,61,62,63].

## 7. Th17 Regulation Mechanisms

Regulation of Th17 cell polarization and functions is primordial for the maintenance of homeostasis and for avoiding chronic inflammatory episodes once a pathogen is cleared. Restricting either IL-17 signaling or de novo Th17 generation are ways to stop exacerbated Th17 immune responses. Ubiquitination of components involved in IL-17A signaling transduction pathway is one of the mechanisms that inhibit expression of IL-17A target genes [4]. Reported molecules implicated in the negative regulation of IL-17 signaling include deubiquitinating enzymes, A20, ubiquitination adaptor proteins, micro-RNA (miR)-23b, and the CCAAT/enhancer-binding protein transcription factors (C/EBPs) [4]. Interestingly, A20 and C/EBPs are induced by IL-17 signaling and can act separately or in combination to repress IL-17A targeted genes.

Inhibition of de novo Th17 generation in mucosal tissues is dependent on the specific cues present in local environment. For example, the presence of high levels of TGF-β and/or retinoic acid promotes the differentiation Foxp3+ Tregs thus shutting down Th17 development [3,192]. IL-2 inhibits Th17 differentiation by activating STAT5 that competes with STAT3 for binding sites across the *il17a* locus [92,93]. In addition, IL-2 can function jointly with TGF-β to induce Tregs. Through the interaction with AhR and c-maf, IL-27 promotes the generation of T regulatory type 1 cells (TR1) producing IL-10 and is dependent on STAT1 expression [23,193]. IL-27 can directly inhibit RORγt expression in both mice and humans [194] thereby blocking the Th17 development. IL-27 has an effect on naive T-cells undergoing Th17 differentiation [23]. Th17-mediated immune responses are well controlled after pathogen clearance. Results generated by the group of Richard Flavell support the concept that once the infection/inflammation is resolved, Th17 cells leave the site of inflammation and migrate towards the duodenum [27]. This trafficking process is mediated by the CCL20-CCR6 axis [175]. Once in the duodenum, Th17 cells are eliminated by unknown mechanisms or acquire features of Tregs (e.g., IL-10 production). These regulatory Th17 cells are immunosuppressive as they have the ability to prevent cell proliferation [27]. Noteworthy, the in situ co-localization potential of Th17 and Tregs via CCR6 and other chemokine receptors [195] as well as the trans-differentiation of Th17 cells into Tregs [85] contribute to the regulation of Th17-mediated inflammatory responses. In summary, the immune host defense employs several mechanisms to avoid deregulation by Th17 cells and maintain homeostasis.

## 8. Natural Th17 Cells

Originally discovered in 2009 with the use of mice models, natural Th17 (nTh17) cells represent a population of IL-17A producing αβ CD4+ T-cells that undergo maturation and functional priming exclusively in the thymus, similar to natural Tregs (nTregs) [196]. The nTh17 cells exit the thymus and enter the periphery where they acquire a memory-like phenotype in spite of being naive in terms of antigen recognition. It has been proposed that these cells respond to tissue injuries prior to the activation of conventional/inducible Th17 cells that are generated following antigen recognition [197]. Both nTh17 and inducible Th17 cells share common characteristics including the expression of RORγt, CCR6, IL-23R, and the production of IL-17A as well as IL-22 [197]. Similar to inducible Th17 cells, nTh17 cells also exhibit specificity for *C. albicans* and play a pathogenic role in autoimmunity [198,199,200]. Nevertheless, nTh17 differ from inducible Th17 cells in their development pathway since they produce IL-17A and IL-22 following Toll-like receptor (TLR) stimulation independently of TCR engagement [197,199]. TGF-β and IL-23/STAT3 axis were reported to be important factors in nTh17 development and survival, whereas IL-6 was found to be dispensable for the expansion of this subset in the periphery [197]. The mechanism regulating the development of nTh17 cells as well as their extended contribution in protective immunity remains to be further investigated. Also, the existence of this subset of Th17 cells in humans remains to be confirmed.

## 9. Th17 Pathogenicity

Pathogenic Th17 cells have been extensively described in the context of autoimmunity as a source of TNF-α and GM-CSF [13,27,83] and were originally discovered in the context of EAE murine models [6,37]. Th1Th17/Th1* cells producing both IL-17A and IFN-γ have been reported as pathogenic cells exacerbating disease pathogenesis [13,84,149,201] (Figure 1A). They are the main tissue-infiltrating CD4+ T-cells in several inflammatory disorders such as rheumatoid arthritis (RA), psoriasis, Crohn’s disease, and multiple sclerosis (MS) [201,202]. Pathogenic Th17 cells induced by IL-23 are responsible for EAE development [4]. In fact, IL-23 has been reported to induce the production of GM-CSF [203], which has been identified as the key factor involved in the EAE onset and an essential component of Th17 pathogenicity [83,203,204]. Recently, several studies performed in mice described the presence of non-pathogenic Th17 cells able to produce IL-10 and displaying immune-suppressive properties limiting tissue inflammation [27,83,205,206,207,208] (Figure 1A). There is experimental evidence supporting the concept that pathogenic Th17 cells can convert into non-pathogenic cells [27]. Furthermore, during the resolution of an immune response, Th17 cells lose their capacity to produce IL-17A as well as the expression of high levels of RORγt [85]. These Th17 cells undergo trans differentiation into TR1 and acquire the ability to express IL-10 and TR1 surface marker LAG-3 [85].

Therefore, accumulating data indicate that Th17 cells homogenously producing IL-17A do not represent end stages of memory T-cell differentiation as they can further gain new effector functions associated with other T-cell lineages (trans-differentiation). The plasticity displayed by Th17 cells broadens their function and therefore once recruited into specific anatomic locations they efficiently promote diversity in host defense [55,126,149].

## 10. The Discovery of Long-Lived Th17 Cells in Cancer

The pathogenic versus non-pathogenic features of Th17 cells in cancer remains controversial and is likely dependent on the type of cancer (Figure 1B) [209]. Th17 cells appear to promote disease progression in hepatocellular carcinoma and gastric cancer [210,211]. Th17 cells were reported to be present in the vicinity of several malignancies including ovarian [212], gastric [213], colorectal [214], breast [215], pancreatic carcinomas [216], and melanomas [214]. Tumors secrete monocyte chemoattractant protein 1 (MCP-1) that attracts monocytes and RANTES that recruits Th17 cells [215]. At the opposite, Th17 cells were reported to provide protective immunity in melanomas and ovarian cancer [217,218,219,220,221]. In melanomas, tumor-specific Th17 cells led to the recruitment on CD8α+ DCs and activation of tumor-specific CD8+ T-cells, which were crucial for the prevention of tumor development [217]. Th17 cells confer protection against ovarian cancer by the recruitment of effector cells through the production of CXCL9 and CXCL10 [219]. The production of IFN-γ by Th17 cells appears essential for an efficient immune response against both melanomas and ovarian cancers [218,219,221]. The role of IFN-γ in Th17 cells-mediating anti-tumor effect also appears to be specific to the type of cancer. Neutralization of IFN-γ in murine models had no impact on the Th17 anti-tumor properties against lung cancer [217]. These striking differences reported in these studies may reflect the type of Th17 cells present in specific cancers. The complete phenotypic and functional characterization of Th17 cells in the context of cancer remain to be investigated.

Interestingly, studies in mice and humans in the context of cancer revealed the existence of Th17 cells with features of a newly characterized population of T lymphocytes with self-renewal properties [220,221], known as stem memory T-cells (TSCM) [222]. These Th17 cells were referred to as long-lived Th17 cells. The TSCM were originally observed in mice in the context of graft-versus-host disease [223] and were later rediscovered in the context of cancer [224,225]. The TSCM were described not only for their robust self-renewal and expansion capacity but also for their ability to be progenitors for effector T-cells as well as central and effector memory T-cells. Therefore, TSCM have characteristics of hematopoietic stem cells [226]. Long-lived Th17 cells were described in mice and in humans as Th17 cells expressing molecules of the Wnt-β catenin signaling axis [220,221], a pathway associated with self-renewal and survival capacity in stem cells [227]. In humans, long-lived Th17 cells express stem cell-related genes such as Nanog, OCT4, Bcl2, Notch, and SOX2 [220]. Survival and apoptosis of human long-lived Th17 cells are controlled by the HIF-1α/Notch/Bcl-2 signaling pathway. Both murine and human long-lived Th17 cells co-express IL-17A and IFN-γ (Th1Th17/Th1* profile) and exhibit a phenotype of terminally differentiated memory T-cells, as reflected by their low level of CD27 expression [220,221]. Adoptive T-cell transfer in mice demonstrated that CD4+ T-cells producing IL-17A are progenitors of long-lived Th17 cells [221]. The discovery that specific Th17 subsets display stem-cell features raise the possibility that such subsets may contribute to diverse pathologies including cancer and HIV.

## 11. Role of Th17 Cells in HIV-1/SIV Pathogenesis

The role of Th17 cells in human and simian immunodeficiency virus (HIV/SIV) pathogenesis is different from autoimmunity and cancer. In fact, HIV/SIV infection induces massive depletion of CD4+ T-cells, mainly Th17 cells, in the gastrointestinal tract [228,229]. The loss of Th17 cells contributes to HIV/SIV pathogenesis [56,230]. Indeed, the loss of Th17 cells in mucosal tissues of SIV-infected primates is associated with increased viremia [231]. Depletion of Th17 cells is considered a major cause of microbial translocation from the gut, a process leading to chronic immune activation and disease progression, as originally shown in primate models studying SIV pathogenesis [232,233] and further confirmed in human studies [234,235]. The paucity of Th17 cells was associated with translocation from the gut of the pathogen *Salmonella typhimurium* [236]. This depletion occurs within weeks following primary infection and persists during the chronic phase of SIV infection [231]. The natural hosts of SIV infection, the sooty mangabeys, conserve their Th17 cells following primary infection and exhibit little immune activation and disease progression [237]. Similarly, human studies reported that the frequency of Th17 cells is preserved in HIV-infected long-term non-progressors [238,239].

Although the complete mechanisms explaining the loss of Th17 cells during HIV/SIV infections are still under investigation, well-established mechanisms include: (i) HIV infection [240,241,242]; (ii) deficient expression of Th17 polarizing cytokines [140,230]; (iii) altered Th17 trafficking into the gut [243,244]; (iv) expansion of Tregs [245,246]; (v) overexpression of factors inhibiting Th17 differentiation program [247]; (vi) depletion of CD103+ DC from the gut [248]; and (vii) depletion of naive Th17 precursors [249,250]. These mechanisms are described in detail here below.

In the gut mucosa, IL-17-producing cells as well as CCR6+ T-cells express at the highest levels the major HIV co-receptor CCR5 [237,251]. The CCR5+ Th17 cells were shown to be preferentially depleted in the gastrointestinal tract of HIV-infected individuals [237]. It was also reported that peripheral blood Th17 cells co-expressing CCR6 and CCR5 are significantly depleted in HIV-infected individuals [241]. While the depletion of gut Th17 cells is accepted in the field, the depletion of blood Th17 cells is controversial. We previously reported decreased frequencies of peripheral blood CCR4+CCR6+ Th17 and CXCR3+CCR6+ Th1Th17 cells in chronically HIV-infected individuals during ART (CI on ART) compared to uninfected controls [240] (Figure 1C). Consistently, studies from El Hed et al. reported a decrease in the frequency of CD4+ T-cells producing IL-17A in HIV-infected individuals compared to uninfected controls [241]. The decrease observed from both studies was minimal in terms of percentages but the results were statistically significant [240,241]. Minimal secretion of IL-17A from CD4+ T-cells was observed in HIV-infected children with detectable viremia [252]. Furthermore, the studies performed by our group and others reported the drastic depletion of CD4+ T-cells producing IL-17A, with the reduction going up to 10-fold [249,253]. Recent studies using CCR6 in the presence or absence of CD161 also confirmed the decrease of Th17 cells in rhesus macaques [254] similar to humans [249]. Nevertheless, Ciccone et al as well as Brenchley et al. reported no depletion of blood Th17 in HIV-infected individuals [237,238].

Although Brenchley et al. found that HIV infection is not predominant in Th17 cells [237], our group and others reported preferential infection of Th17 compared to Th1 cells in vitro and ex vivo [240,241,242]. This difference could be explained by the fact that Brenchey et al. quantified HIV-DNA expression in cells exhibiting Th17 effector functions (IL-17A) to make their observation [237], whereas studies by our group and others used surface markers for Th17 identification [240,241,242], including CCR6, CCR4, and CXCR3 [152,155]. Our group previously demonstrated that memory CCR6+ T-cells harbored higher levels of integrated HIV-DNA compared to the CCR6− counterpart population in HIV-infected untreated individuals [240]; also, exposure to the gut-homing inducer retinoic acid selectively increases HIV permissiveness in CCR6+ T-cells [255]. The preferential infection of Th17 cells was also confirmed in SIV-infected rhesus macaques [256]. Furthermore, recent studies by Stieh D et al. demonstrated that CCR6+RORγt+ Th17 cells are the very first targets of SIV following infection in the endocervix of rhesus macaques [257]. Consistently, we recently demonstrated that HIV-DNA reservoirs preferentially persist in colon and blood CCR6+ T-cells of ART-treated individuals [258]. Together, these findings suggest that the permissiveness of Th17 cells to HIV/SIV infection is a major cause for their depletion.

The molecular mechanism explaining the preferential permissiveness of Th17 cells to HIV infection is starting to be elucidated. Our group demonstrated that Th17 cells express high levels of NF-κB and a large panel of HIV dependency factors [259,260]. Another recent study linked the susceptibility of Th17 cells to HIV to their inability to express RNase a superfamily proteins, including RNase 6, shown to inhibit viral replication [261]. Most recently, we demonstrated that HIV preferentially targets gut-homing CCR5+ Th17 cells via mTOR-dependent mechanisms and demonstrated the ability of retinoic acid, a gut-homing modulator, to induce mTOR expression and phosphorylation [251]. mTOR increases in HIV permissiveness by acting at various levels of the viral cycle including CCR5-mediated entry [262] and/or transcription [251,263]. Similar to gut-homing Th17 cells, Th17 cells from the female reproductive tract co-express CCR5 and CD90 and are susceptible to HIV infection [257,264]. Interestingly, only a fraction but not all Th17 cells are infected in vitro [181,241,242]). Also, Th17 appear to be preferentially depleted from the gut but not from the lung [237]. The possibility that Th17 cells are not depleted in the lungs during HIV infection remains intriguing considering the fact that the lungs may represent an important site of HIV replication and persistence, as documented in macrophages [265,266,267].

The lack of Th17 polarizing cytokine in the local environment may explain a Th17 deficit in HIV-infected individuals. While levels of IL-6 and TGF-β1 are relatively high during HIV and SIV infection [230], plasma IL-21 levels are diminished in AIDS patients [268]. The decreased frequency of IL-21-producing CD4+ T-cells is directly associated with the loss of Th17 cells in the blood and rectal mucosa of SIV-infected primates [140]. Of particular importance, treatment with recombinant IL-21 in SIV-infected primates leads to increased Th17 frequency [140,142]. Also, IL-21 supplementation combined with pro-antibiotics in SIV-infected macaques treated with antiretroviral therapy (ART) led to an increase in the frequency of poly-functional Th17 cells capable of producing IL-2, IL-22, and TNF-α [269]. Thus, the IL-21 deficit may represent one cause for Th17 paucity during HIV/SIV infections that could be counteracted therapeutically.

Impairment in the trafficking of gut-homing cells may also be involved the incomplete reconstitution of mucosal immunity. Mavinger et al. showed that gut-homing CD4+ T-cells expressing CCR9 and integrin β7, including Th17 cells, failed to migrate into the intestine and accumulated in the peripheral blood of chronically-infected patients receiving ART [243]. This altered trafficking was due to decreased expression of the CCR9 ligand CCL25 in the intestines of these HIV-infected individuals. The paucity of CD4+CCR9+β7+ T-cells in the gut was linked to microbial translocation and systemic immune activation [243]. Similarly, other reports revealed a reduction in CCL20 levels in the gut of SIV-infected primates [236,245], thereby preventing migration of circulating CCR6+ Th17 cells. Recent studies further demonstrated the impairment of the CCR6-CCL20 axis in treated HIV-infected individuals resulting in the inability of the Th17 cells to migrate in intestinal mucosa [244,270]. The mechanisms explaining this alteration may include (i) increased frequency of CCR6− Tregs that blunt the production of CCL20 from the enterocytes [244] and (ii) an impaired migration potential of Th17 cells due to inefficient actin polymerization [270].

The depletion of Th17 cells was also shown to be associated with expansion of Tregs during HIV/SIV infections [271]. The expression of the Treg master regulator Foxp3 is increased in untreated HIV-infected individuals [245,272]. The alteration of the Th17/Treg ratio is due to the accumulation of byproducts of tryptophan metabolism [246]. Tryptophan catabolites promote the expression of FoxP3 in T-cells resulting in the down regulation of RORγt and subsequent inhibition of Th17 generation [273,274]. Also, tryptophan deprivation can enhance Treg development to the detriment of Th17 differentiation. The catabolism of tryptophan is mediated by the enzyme indoleamine 2,3-dioxygenase 1 (IDO-1), expressed by mucosal DC; IDO expression was found up regulated during HIV/SIV infections [245,246,275]. Indeed, Favre et al. discovered that DCs produce high levels of IDO-1; this promotes the generation of T regulatory (Treg) cells as opposed to Th17 cells and thereby shifts the Th17/Treg ratio in the gut [245,246]. Interestingly, the Th17/Treg ratio is maintained in the natural host of SIV [245] and also in HIV-infected individuals than control viral replication and preserve normal CD4 counts in the absence of ART, also known as elite controllers [276].

Overexpression of negative regulators of the Th17 differentiation program may also explain the depletion of Th17 cells during HIV infection. Suppressor of cytokine signaling-3 (SOCS3), protein inhibitor of activated STAT3 (PIAS3), and protein tyrosine phosphatase (SHP-2) are negative regulators of STAT3 [230]. The expression of SOCS3 mRNA is increased in HIV-infected individuals. The high levels of SOCS3 in mucosal tissues were associated with the increased permissiveness to HIV infection [277]. In the context of SIV-pathogenesis, the up-regulated expression of SOCS3, PIAS3, and SHP-2 was correlated to the suppression of IL-17 [247]. The suppression of Th17 cells in these animals led to an increase of soluble CD14, a marker of microbial translocation [247]. Whether negative regulators of Th17 polarization are also increased in cells from HIV-infected subjects remains unknown.

Other mechanisms involved in Th17 deficiency include the loss of CD103+ DCs that was described to be associated with the reduced frequency of Th17 cells in the gut of HIV-infected individuals [248]. In addition, our group demonstrated that naive CD4+ T-cells from chronically HIV-infected individuals on ART are impaired in their ability to generate Th17 cells compared to uninfected individuals [249]. This deficit was associated with the depletion of Th17 precursors in that were identified as naive Tregs (nTregs: CD25highCD127−FoxP3+) and naive T-cells expressing CD25 and CD27 but lacking the expression of FoxP3, called double positive cells (DP) [249]. The decrease of these Th17 precursors was associated with the reduced proportion of Th17 memory cells in our cohort [249]. Noteworthy, nTregs and DPs harbored higher levels of integrated HIV-DNA compared to classical naive CD25-CD127-FoxP3- T-cells [249]. Consistently, another study demonstrated that HIV infection is associated with the depletion of a T-cell population derived from naive CCR6+ precursors, expressing IL-17A, FOXP3, and CD25 (IL-17A-Tregs) in HIV-infected subjects [250]. Whether the naive CCR6+ T-cell precursors are depleted from HIV-infected patients remains to be determined. If proven to be the case, it would further explain the incapacity of the host immune system to replenish Th17 cells during HIV infection.

The ability of ART in restoring the gut and peripheral Th17 cell counts remains controversial. Kim et al. reported that short-term ART restores the numbers of sigmoid colon Th17 cells but not their function during chronic infection [188], even with ART intensification [278]. Furthermore, studies by Ciccone et al. as well as Chege et al. reported similar frequencies of gut and/or peripheral blood Th17 cells between ART-treated HIV-infected and uninfected individuals [189,238]. Nevertheless, several groups reported that ART only led to partial restoration of Th17 cells [243,279,280]. The mechanism involved may include the alteration in the trafficking of gut-homing cells via the CCR6-CCL20 and/or the CCR9-CCL25 axis [243,244]. Increased frequency of total CD4+ T-cells in the gastrointestinal tract following treatment was correlated with the partial restoration of Th17 cells [186]. Considering the fact that ART initiated during chronic infection fails to completely restore Th17 cells [186], even when ART is intensified with raltegravir (integrase inhibitor) and maraviroc (CCR5-mediated entry inhibitor) [278], the search for additional strategies to restore Th17 cells in chronically-infected individuals is ongoing. Indeed, treatment with probiotics along with ART in SIV-infected pigtail macaques was reported to increase the levels of IL-23, which was accompanied with the enhancement of Th17 cells when compared to animals that were only treated with ART [235]. Other studies showed that the addition of prebiotics and probiotics leads to a reduction of microbial translocation and chronic immune inflammation, thus improving the health of patients [281]. Nevertheless, certain studies did not succeed in demonstrating the benefits of probiotics on HIV infection [230]. Nevertheless, Ortiz et al. recently reported that combined administration of IL-21 and probiotics led to Th17 expansion and a decrease in markers of microbial translocation in SIV-infected pigtail macaques treated with antiretroviral drugs [269]. Future investigations should clarify the potential beneficial use of probiotics in the context of HIV infection.

The time of ART initiation may be critical in the maintenance versus depletion of the Th17 population. Indeed, an important study performed by Schuetz et al. using a unique cohort of individuals at high risk of HIV acquisition demonstrated that only the administration of ART on during the very early acute phases of HIV infection (Fiebig I and II) prevented the depletion of mucosal Th17 cells [190]. In contrast, the administration of ART at Fiebig III stage allowed the restoration of only the frequency but not the poly-functionality of Th17 cells [190]. However, such early ART interventions remain almost utopical considering the fact that HIV diagnosis typically occurs during late stages of primary infection if not during the chronic phase. Therefore finding alternatives to restore Th17 cells in chronically HIV-infected subjects remains a major research priority.

## 12. Pathogenic Versus Non-Pathogenic Th17 Cells during HIV/SIV Infections

The molecular determinants of Th17 pathogenicity appear to be disease-specific. Even in the context of autoimmunity, the pathogenic features of Th17 cells are distinct in different autoimmune diseases. While the increased presence of Th17 cells is deleterious in MS and psoriasis [4,202], the absence of cells producing IL-17A leads to the exacerbation of Crohn’s disease [34]. Indeed, very recent studies demonstrated the critical role of IL-17A in the maintenance of barrier function at epithelial cell level, which may influence the outcome of IBD pathogenesis in patients [33,34]. In the context of HIV infection, the loss of Th17 cells is detrimental. The Th17 depletion leads to microbial translocation, chronic immune activation, and the emergence of opportunistic infections including candidiasis [56,235,282]. Pathogenic Th17 cells during HIV infection can be described as cells that are permissive to HIV replication. Indeed, our group demonstrated that the Th17 surface marker CCR6 identifies CD4+ T-cells highly permissive to HIV infection in vitro and enriched in HIV-DNA in infected individuals, as opposed to CCR6− cells [240,251,255,258,259,260]. Other groups confirmed these findings [241,242]. In addition, studies performed in rhesus macaques reported that the CCR6+CD4+ T-cells expressed the highest levels of SIV-RNA [254]. The fact that only a fraction of Th17 cells support HIV replication points to the existence of HIV resistant Th17 cells that may be considered non-pathogenic. The depletion of Th17 cells in the gut but not in the lungs of HIV-infected individuals [237], raises the possibility that Th17 cells infiltrating the lungs are resistant to HIV and therefore non-pathogenic. Alternatively, these cells may be infected and exhibit long-lived features and therefore they may be pathogenic by contributing to HIV persistence.

While the phenotypic profile of pathogenic versus non-pathogenic Th17 cells in the context of HIV remains yet unknown, in autoimmunity, MDR1 was identified as a surface marker of human pathogenic IL-17A-producing CD4+ T-cells [84]. Precisely, MDR1 is highly expressed in CCR6+CXCR3+ Th1Th17 cells compared to CCR6+CCR4+ Th17 cells [84]. Our group has demonstrated that both Th17 and Th1Th17 cells are permissive to HIV infection [181,240]. One of the reasons why Th1Th17 may be considered more pathogenic in the context of HIV infection is that these cells express the highest levels of CCR5 and integrin β7 and produce TNF-α [240]. Based on the differential expression of CXCR3, CCR6, and CCR7, central memory CXCR3+CCR6+ T-cells, that include the Th1Th17 cells, were recently demonstrated to preferentially harbor HIV-DNA in HIV-infected individuals receiving ART [283]. Results from our group demonstrated that both central memory Th17 and Th1Th17 cells express higher levels of integrated HIV-DNA compared to their Th1 and Th2 counterparts [258]. Whether MDR1 identifies Th17 cells permissive to infection and/or resistant to antiretroviral drugs and thus represent a marker for pathogenic Th17 cells in the context of HIV-1 infection remains to be investigated. The possibility that Th17 cells expressing MDR-1 escape antiretroviral drugs and contribute to HIV reservoir persistence under ART is supported by published evidence that certain antiretroviral drugs are MDR1 substrates [284,285].

The definition of pathogenic Th17 cells in the context of HIV implies the characterization of distinct Th17 subsets. Recent studies by our group and others described that pathogenic Th17 cells can be long-lived and contribute to HIV reservoir persistence [181,286]. Studies by Sun et al. demonstrated that HIV-DNA reservoirs in the Th17 and Th1Th17 cells remained stable for a long period (more than 50 months) in HIV-infected subjects undergoing ART [286], indicating the important contribution of these subsets in HIV persistence. Also, phylogenetic analysis of HIV reservoirs indicated the long-termed persistence of identical viral sequences preferentially expressed in these two Th17 subsets. In addition to Th17 and Th1Th17 subsets, our group identified two new Th17-polarized subsets, CCR6+DN and CCR6+DP, and explored their permissiveness HIV infection in vitro and in ART-treated individuals [181]. Both CCR6+DN and CCR6+DP subsets were susceptible to HIV infection in vitro and harbored relatively high levels of integrated HIV-DNA in HIV-infected subjects undergoing ART [181]. Interestingly, HIV reactivation was preferentially observed in CCR6+DN cells from chronically HIV-infected individuals. In contrast to the other CCR6+ subsets that were depleted in frequency and counts, the frequency/counts of CCR6+DN cells were preserved among the HIV-infected individuals on ART (Figure 1C). Whether the persistence of replication-competent HIV reservoirs in CCR6+DN cells contribute to HIV disease progression remains to be determined. One of the mechanisms explaining the preservation of CCR6+DN cells may include the expression of TSCM-specific molecular markers [181]. Indeed, our study indicated that the CCR6+DN cells exhibited long-lived Th17 features, which may help them counteract the HIV cytopathic effects. The concept that long-lived Th17 cells contribute to HIV persistence under ART, as supported by our findings [181] and others [286], adds to the complexity of the Th17 pathogenicity concept and position these cells as an important barrier to HIV eradication. Furthermore, CCR6+DN cells were shown to express markers of Tfh, a subset of cells recently characterized as major HIV reservoir during ART in lymph nodes and peripheral blood [287]. The relative contribution of CCR6+DN cells amongst the Th17 subsets in terms of HIV persistence remains to be determined in larger HIV-infected cohorts. The fact that CCR6+DN cells are preserved in chronically HIV-infected individuals receiving ART suggest that these subsets represent major contributors of HIV dissemination and persistence within the Th17 lineage. The role of CCR6+DN as Tfh promoting B-cell maturation also remains to be explored. The evidence gathered in Wacleche et al. indicate that CCR6+DN cells contribute to HIV pathogenesis in a distinct manner compared to Th17 and Th1Th17 cells [181]. Thus, the use of CCR6, CCR4, and CXCR3 as surface markers characterizing the Th17 subsets may serve as a tool to identify different types of pathogenic Th17 cells and requires further investigations in different pathological conditions including HIV.

The identification of HIV-permissive pathogenic Th17 cells points to the existence of non-pathogenic Th17 cells resistant to HIV infection. Although HIV-resistant Th17 cells have not yet been identified/characterized, in vitro studies from our group and others demonstrating that only small fractions of Th17 subsets are infected with HIV [181,240,241,242,258], support the existence of such non-pathogenic Th17 subsets. Investigations on HIV-resistant Th17 subsets are important and may lead to the identification of molecular mechanisms insuring Th17 cell protection from infection, mechanisms that could be exploited therapeutically.

The majority of studies aimed at the identification of molecular determinants of HIV permissiveness/resistance in Th17 cells were conveniently performed on cells derived from the blood [240,241,242,251,255,258,259,260,261]. Only few studies explored the mechanisms of HIV permissiveness/resistance in cells infiltrating the gut [184,185,251]; such studies on tissue infiltrating Th17 cells are the most relevant for finding ways to prevent HIV replication/persistence in this unique CD4+ T-cell pool.

## 13. Conclusions

Th17 lymphocytes are essential for the clearance of extracellular pathogens including fungi and bacteria at skin and mucosal barrier surfaces. Th17 differentiation is complex and implies a series of positive and negative regulators that can be targeted therapeutically. The differential expression of the chemokine receptors CCR6, CCR4, and CXCR3 identifies unique subsets of IL-17A-producing cells, including the well-characterized Th17 and Th1Th17 cells [155], as well as the CCR6+DN and CCR6+DP subsets recently identified by our group (Figure 1C,D) [181]. These four subsets express a common Th17-specific molecular signature but also unique sets of transcripts that may render them functionally distinct. The Th17 lineage is known to be involved in several pathologies including autoimmunity, cancer, and HIV. The exact role of these four Th17 subsets in the pathogenesis of specific diseases remains to be further investigated. Although the definition of pathogenic Th17 cells has been extensively characterized in the context of autoimmunity, the concept of pathogenic versus non-pathogenic Th17 cells in the context of HIV infection is gaining interest in our search for novel therapeutic approaches toward HIV remission or cure. One undeniable fact is that Th17 cells play a beneficial role during HIV infection by their capacity to maintain barrier surface integrity and homeostasis, mainly in the gut. However, studies by our group and others demonstrated that HIV takes advantage of the metabolic state and the long-lived features of Th17 cells for its dissemination and persistence. The discovery of this novel level of heterogeneity among Th17 subsets implies that each subset may play a unique role in HIV pathogenesis, likely in a tissue- and/or antigen-specific manner. Future investigations are needed for the detailed identification/characterization of pathogenic versus non-pathogenic Th17 cells in the context of HIV infection, probably via genome-wide transcriptional profiling at the single-cell level on Th17 cells infiltrating mucosal sites, as performed in the context of autoimmunity [288]. This strategy will likely unveil new molecular determinants of HIV pathogenicity that may be targeted in Th17-specific therapeutic interventions aimed at preserving Th17-mediated immunological functions by preventing HIV infection/persistence in these cells.
